# Exact solutions and bounds for network SIR and SEIR models using a rooted-tree approximation

**DOI:** 10.1007/s00285-022-01854-9

**Published:** 2023-01-10

**Authors:** Cameron Luke Hall, Bram Alexander Siebert

**Affiliations:** 1 University of Bristol, Bristol, UK; 2 University of Limerick, Limerick, Ireland

**Keywords:** Primary: 92D30, 60J28, Secondary: 05C05, 05C90, 34C12, 37N25

## Abstract

In this paper, we develop a new node-based approximate model to describe contagion dynamics on networks. We prove that our approximate model is exact for Markovian SIR (susceptible-infectious-recovered) and SEIR (susceptible-exposed-infectious-recovered) dynamics on tree graphs with a single source of infection, and that the model otherwise gives upper bounds on the probabilities of each node being susceptible. Our analysis of SEIR contagion dynamics is general to SEIR models with arbitrarily many classes of exposed/latent state. In all cases of a tree graph with a single source of infection, our approach yields a system of linear differential equations that exactly describes the evolution of node-state probabilities; we use this to state explicit closed-form solutions for an SIR model on a tree. For more general networks, our approach yields a cooperative system of differential equations that can be used to bound the true solution.

## Introduction

Network-based models have been used extensively to describe the spread of a contagious state (such as an infectious disease) through a population via the connections between individuals (Danon et al. 2011; Kiss et al. 2017; Miller and Kiss 2014; Pastor-Satorras et al. 2015). In many contagion models on networks, each node represents an individual and each edge represents a contact or connection that facilitates the spread of contagion between nodes. At any given time, each node has a state—*e.g.*, susceptible, infectious, or recovered in the classic SIR model (Newman 2018; Pastor-Satorras et al. 2015)—and the node states evolve over time according to the rules that constitute the contagion model. In many such models, node state evolution is probabilistic and occurs over continuous time; in these cases, the spread of contagion through the network is a continuous-time discrete-space stochastic process where the state space is the set of states for all nodes in the network.

One challenge with stochastic network contagion models is to determine the node state probabilities as functions of time. Even for very simple contagion models, this is difficult on large networks because the node states do not evolve independently. In the most general case, node state probabilities can only be determined exactly from network state probabilities, which in turn can only be determined exactly by solving the master equations for the stochastic process. Since the size of the state space increases geometrically with the number of nodes, this is not computationally feasible on any but the smallest networks.

Instead, various methods have been developed for estimating—and, in some cases, bounding—node state probabilities in network contagion models. The simplest of these is the node-based mean field approximation (Pastor-Satorras et al. 2015); this approximation is also called the first-order model (Newman 2018), the individual-based model (Kiss et al. 2017), or the *N*-intertwined mean field approximation (Simon and Kiss 2018; Van Mieghem et al. 2009). In this approach, node state probabilities are assumed to be independent of each other, so that joint probabilities can be expressed as the product of individual node state probabilities. While this is a useful assumption that closes the evolution equations for node state probabilities, it is not perfectly accurate. In reality, the states of neighbouring nodes are positively correlated: *e.g.*, the neighbours of a susceptible node are more likely to be susceptible than would be expected from assuming independence (Donnelly 1993). As a result, the node-based mean field approximation applied to standard contagion models will typically overestimate rates of infection and hence underestimate the probability that a given node is susceptible.

Two other approaches used to estimate and bound node state probabilities are the pair-based approximation (Cator and Mieghem 2012; Pastor-Satorras et al. 2015) and the message-passing approximation (Karrer and Newman 2010). To develop the pair-based approximation, Cator and Mieghem (2012) introduced variables for the joint probabilities of the states of neighbouring nodes and they derived evolution equations for these probabilities using a closure approximation to exclude the dependence on higher-order moments. To develop the message-passing approximation, Karrer and Newman (2010) considered the directed edges of the network and developed expressions for the probabilities that infection has not yet been transmitted along each edge.

While these two approaches are conceptually very different, Wilkinson and Sharkey (2014) showed that they are equivalent for Markovian SIR dynamics. Pair-based and message-passing approximations are more computationally demanding than node-based approximations but are generally more accurate than the node-based mean field approximation. When the underlying network is a tree, both approaches yield exact results for the SIR model (Karrer and Newman 2010; Sharkey et al. 2015).

In this paper, we develop and analyse a new approximate model of network contagion that can be applied to Markovian SIR and SEIR (susceptible-exposed-infectious-recovered) contagion models, including SEIR models with multiple distinct exposed states. The approximation we derive is a ‘node-based’ approximation; it takes the form of a closed system of differential equations for node state probabilities. As such, our approximation has a similar level of computational complexity to the node-based mean field approximation and is considerably simpler than the pair-based or message-passing approximations.

We refer to our approximation as the ‘rooted-tree approximation’ because it yields exact results on trees with a single initially-infectious node. This contrasts with both the node-based mean field model, which can never give exact results, and the pair-based and message-passing approximations, which give exact results on any tree regardless of the number of initially-infectious nodes (Karrer and Newman 2010; Sharkey et al. 2015). The exact differential equations obtained using our approximation are very simple and lead to explicit closed-form solutions for node state probabilities on rooted trees. We believe that these explicit solutions have not previously been reported.

On other networks (non-trees or trees with multiple initially-infectious nodes), we prove that the rooted-tree approximation gives *upper* bounds on the probabilities that nodes are susceptible. This contrasts with the other approximations described above, which give *lower* bounds on the probabilities that nodes are susceptible; this lower bound result is generally understood to hold for node-based mean field approximation of SIR models (Cator et al. 2018; Cator and Mieghem 2014; Donnelly 1993) and has been proved for node-based mean field approximation of SIS models (Donnelly 1993; Simon and Kiss 2018) and for pair-based/message-passing approximation of SIR models (Karrer and Newman 2010; Wilkinson and Sharkey 2014)

The development of our approximation exploits the fact that neither the SIR nor SEIR models permit the possibility of reinfection. In the case of an SIR model on a tree with a single initially-infectious node, this enables us to formulate an exact expression for the rate of infection in terms of the probabilities that nodes are susceptible. For other networks and initial conditions, a similar approach enables us to formulate a cooperative system of differential equations where the approximate rate of infection is a lower bound on the true rate of infection. This enables us to use the methods developed by Simon and Kiss (2018) to prove that our approach yields upper bounds on the probablilities that nodes are susceptible.

Our main contribution in this paper can be summarised as the rooted-tree approximation systems given in (28) and (64) for SIR and SEIR models respectively. In Sect. 2, we develop (28) for SIR models and prove that it is exact on rooted trees and otherwise yields an upper bound on the probability of being susceptible. In Sect. 3, we repeat this analysis for SEIR models to develop (64). Finally, in Sect. 4, we discuss the merits and limitations of our approach and make comparisons with other theoretical approaches to network contagion. We conclude by offering avenues for further exploration and extension of the rooted-tree approximation.

## Rooted-tree approximation for the SIR model

### Preliminaries

Let $\{{\textbf{X}}(t)\}$ represent the stochastic process for network contagion dynamics on a network of $N$ nodes. Any realisation of this process can be represented as a time-dependent $N$-dimensional vector of node states, ${\textbf{X}}(t)$, so that $X_{k}(t)$ gives the state of the $k$th node at time *t*. Following various other authors (Sharkey et al. 2015; Sharkey and Wilkinson 2015; Simon and Kiss 2018; Wilkinson and Sharkey 2014), we use angle brackets to indicate probabilities. Specifically, we define $\langle S_{{k}} \rangle (t) = P\left[ X_k(t) = \text {S}\right]$ to be the probability that node $k$ is susceptible at time *t*, we define $\langle I_{j} S_{k} \rangle (t) = P\left[ X_j(t) = \text {I} \cap X_k(t) = \text {S}\right]$ to be the probability that node $j$ is infectious and node $k$ is susceptible at time *t*, and we define other probabilities and joint probabilities similarly.

In this section, we focus on the standard network SIR model as described in Newman (2018) and elsewhere. At any time, each node can either be susceptible (S), infectious (I) or recovered (R) and node states change over time according to a Markovian process. Susceptible nodes in contact with infectious nodes become infected at rate $\lambda _{}$; that is, the probability that a susceptible node in contact with an infectious node becomes infectious in the next $\Delta t$ is given by $\lambda _{} \Delta t + o(\Delta t)$. Infection rates are taken to be additive over neighbours, so that additional infectious neighbours will increase the probability that a susceptible node becomes infectious in a given $\Delta t$. Infectious nodes recover at rate $\gamma _{}$ regardless of the states of their neighbours.

As a further generalisation, we assume that $\lambda _{}$ can depend on the associated directed edge, and that $\gamma _{}$ can depend on the associated node. Thus, we assume that the rate of infection can depend on the nodes involved and that the rate of recovery from infection can vary from node to node. We represent this using subscripts, so that $\lambda _{k \leftarrow j}$ is the rate at which node $k$ becomes infected given that node $k$ is susceptible and node $j$ is infectious, and $\gamma _{k}$ is the rate at which node $k$ would recover given that it is currently infectious.

With this notation, the following is an exact description of node probability dynamics for an SIR model on a network:

1a $$\begin{aligned} \frac{\textrm{d} {\langle S_{{k}} \rangle }}{\textrm{d} {t}}&= - \sum _{j \in {\mathcal {N}}(k)} \lambda _{k \leftarrow j} \langle I_{j} S_{k} \rangle , \end{aligned}$$

1b $$\begin{aligned} \frac{\textrm{d} {\langle I_{k} \rangle }}{\textrm{d} {t}}&= \sum _{j \in {\mathcal {N}}(k)} \lambda _{k \leftarrow j} \langle I_{j} S_{k} \rangle - \gamma _{k} \langle I_{k} \rangle , \end{aligned}$$

1c $$\begin{aligned} \frac{\textrm{d} {\langle R_{k} \rangle }}{\textrm{d} {t}}&= \gamma _{k} \langle I_{k} \rangle , \end{aligned}$$

where ${\mathcal {N}}(k)$ represents the set of upstream neighbours of node $k$ (*i.e.*, the set of nodes $j$ for which $\lambda _{k \leftarrow j}$ is nonzero).

### Exact SIR dynamics on a rooted tree

Consider the case where the underlying network is a tree and where a single node is infectious at $t = 0$ and all other nodes are susceptible. We assign the the label $k= 0$ to the initially-infectious node and identify it as the root of the tree. We will use the term ‘rooted tree’ throughout our analysis (including for SEIR models) to refer to a tree where there is a unique node that is not in a susceptible or recovered state at $t = 0$. For any other node $k\ne 0$, it is possible to identify a unique parent node $p(k)$ as the neighbour of $k$ that lies between node $k$ and the root. Since all infection spreads from the root node it follows that node $k$ can only be infected by node $p(k)$. This enables us to simplify our notation and analysis in this section: we define $\lambda _{k} = \lambda _{k \leftarrow p(k)}$ as the rate at which node $k$ is infected by its parent node, and we omit the sums in equations (1a) and (1b).

Thus, the evolution of node state probabilities on a rooted tree is given by

2a $$\begin{aligned} \frac{\textrm{d} {\langle S_{{k}} \rangle }}{\textrm{d} {t}}&={\left\{ \begin{array}{ll} 0, &{} k= 0, \\ -\lambda _{k} \langle I_{p(k)} S_{k} \rangle , &{} k\ne 0; \end{array}\right. } \end{aligned}$$

2b $$\begin{aligned} \frac{\textrm{d} {\langle I_{k} \rangle }}{\textrm{d} {t}}&={\left\{ \begin{array}{ll} - \gamma _{k} \langle I_{k} \rangle , &{} k= 0, \\ \lambda _{k} \langle I_{p(k)} S_{k} \rangle - \gamma _{k} \langle I_{k} \rangle , &{} k\ne 0; \end{array}\right. } \end{aligned}$$

2c $$\begin{aligned} \frac{\textrm{d} {\langle R_{k} \rangle }}{\textrm{d} {t}}&= \gamma _{k} \langle I_{k} \rangle . \end{aligned}$$

These equations need to be solved subject to initial conditions

3 $$\begin{aligned} \langle S_{{k}} \rangle (0)&= {\left\{ \begin{array}{ll} 0, &{} k=0, \\ 1, &{} k\ne 0; \end{array}\right. }, \quad \langle I_{k} \rangle (0) = {\left\{ \begin{array}{ll} 1, &{} k=0, \\ 0, &{} k\ne 0; \end{array}\right. }, \quad \langle R_{k} \rangle (0) = 0. \end{aligned}$$

This system of equations is not closed; in order to construct a node-based model of contagion dynamics, we need expressions for the pair probabilities $\langle I_{p(k)} S_{k} \rangle$ in terms of the node state probabilities. The analysis below shows how this can be achieved exactly on a rooted tree.

Consider any node $k\ne 0$. The law of total probability gives

4 $$\begin{aligned} \langle S_{{k}} \rangle = \langle S_{p(k)} S_{k} \rangle +\langle I_{p(k)} S_{k} \rangle + \langle R_{p(k)} S_{k} \rangle \end{aligned}$$

Since infection can only spread from node $p(k)$ to node $k$ and not *vice versa*, we find that $X_{p(k)} = \text {S}$ implies $X_{k} = \text {S}$ (*i.e.*, if the parent of node *k* is susceptible then node *k* must also be susceptible). Hence, $\langle S_{p(k)} S_{k} \rangle = \langle S_{{p(k)}} \rangle$ and (4) can be rearranged as

5 $$\begin{aligned} \langle I_{p(k)} S_{k} \rangle = \langle S_{{k}} \rangle - \langle S_{{p(k)}} \rangle - \langle R_{p(k)} S_{k} \rangle . \end{aligned}$$

This indicates that an expression for $\langle R_{p(k)} S_{k} \rangle$ in terms of node state probabilities could be used to obtain an expression for $\langle I_{p(k)} S_{k} \rangle$ in terms of node state probabilities.

We note that the only way to achieve a state where $X_{p(k)} = \text {R}$ and $X_{k} = \text {S}$ is for node $p(k)$ to recover while node $k$ is susceptible. Once such a state is achieved, it will persist permanently since node $p(k)$ will remain recovered and node $k$ cannot become infected except via node $p(k)$. Expressed mathematically, this means that

6 $$\begin{aligned} \frac{\textrm{d} {\langle R_{p(k)} S_{k} \rangle }}{\textrm{d} {t}} = \gamma _{p(k)} \langle I_{p(k)} S_{k} \rangle , \qquad k\ne 0, \end{aligned}$$

which can be rearranged using (2a) to yield

7 $$\begin{aligned} \frac{\textrm{d} {\langle R_{p(k)} S_{k} \rangle }}{\textrm{d} {t}} = - \frac{\gamma _{p(k)}}{\lambda _{k}} \frac{\textrm{d} {\langle S_{{k}} \rangle }}{\textrm{d} {t}}, \qquad k\ne 0. \end{aligned}$$

Integrating (7) and applying the initial conditions $\langle R_{p(k)} S_{k} \rangle (0) = 0$ and $\langle S_{{k}} \rangle (0) = 1$ for $k\ne 0$, we find that $\langle R_{p(k)} S_{k} \rangle = \frac{\gamma _{p(k)}}{\lambda _{k}} - \frac{\gamma _{p(k)}}{\lambda _{k}} \langle S_{{k}} \rangle$.

Substituting this into (5) then yields

8 $$\begin{aligned} \langle I_{p(k)} S_{k} \rangle = \frac{\lambda _{k} +\gamma _{p(k)}}{\lambda _{k}} \langle S_{{k}} \rangle - \langle S_{{p(k)}} \rangle - \frac{\gamma _{p(k)}}{\lambda _{k}}, \qquad k\ne 0. \end{aligned}$$

Equation (8) gives an expression for $\langle I_{p(k)} S_{k} \rangle$ purely in terms of the node state probabilities $\langle S_{{k}} \rangle$ and $\langle S_{{p(k)}} \rangle$. Substituting into system (2), we obtain the following closed system for the node state probabilities:

9a $$\begin{aligned} \frac{\textrm{d} {\langle S_{{k}} \rangle }}{\textrm{d} {t}}&= {\left\{ \begin{array}{ll} 0, &{} k= 0, \\ -(\lambda _{k}+\gamma _{p(k)}) \langle S_{{k}} \rangle + \lambda _{k} \langle S_{{p(k)}} \rangle + \gamma _{p(k)}, &{} k\ne 0; \end{array}\right. } \end{aligned}$$

9b $$\begin{aligned} \frac{\textrm{d} {\langle I_{k} \rangle }}{\textrm{d} {t}}&= {\left\{ \begin{array}{ll} - \gamma _{k} \langle I_{k} \rangle , &{} k= 0, \\ (\lambda _{k}+\gamma _{p(k)}) \langle S_{{k}} \rangle - \lambda _{k} \langle S_{{p(k)}} \rangle - \gamma _{p(k)} - \gamma _{k} \langle I_{k} \rangle , &{} k\ne 0; \end{array}\right. } \end{aligned}$$

9c $$\begin{aligned} \frac{\textrm{d} {\langle R_{k} \rangle }}{\textrm{d} {t}}&= \gamma _{k} \langle I_{k} \rangle . \end{aligned}$$

This system can be solved subject to the initial conditions in (3) to yield an exact representation of node state probabilities on a rooted tree.

### Closed form solutions

The system in (9) is amenable to further analysis leading to explicit closed form solutions. We observe that the differential equations in (9) are all linear and have constant coefficients. Moreover, the system is partially decoupled: the equations for $\frac{\textrm{d} {\langle S_{{k}} \rangle }}{\textrm{d} {t}}$ are independent of $\langle I_{k} \rangle$ and $\langle R_{k} \rangle$, the equations for $\frac{\textrm{d} {\langle I_{k} \rangle }}{\textrm{d} {t}}$ are independent of $\langle R_{k} \rangle$, and all equations for node state probabilities at a given node are independent of the states of the node’s children and siblings.

It follows that the differential Eq in (9) can be solved sequentially using standard methods. To achieve this, we first reassign the node labels so that $p(k) < k$ for all $k$; this can be done by taking node 0 as the root and applying either a breadth-first search algorithm or a depth-first search algorithm (Biggs 2002). With this reordering, we first note that equation (9a) is trivial when $k= 0$ and yields the solution $\langle S_{{0}} \rangle (t) = 0$. Next, we find that if $k> 0$ then Eq (9a) takes the form

10 $$\begin{aligned} \frac{\textrm{d} {\langle S_{{k}} \rangle }}{\textrm{d} {t}} + (\lambda _{k}+\gamma _{p(k)}) \langle S_{{k}} \rangle = \lambda _{k} \langle S_{{p(k)}} \rangle + \gamma _{p(k)} \end{aligned}$$

Since the reordered node labels satisfy $p(k) < k$, we observe that the right hand side of (10) involves a function $\langle S_{{j}} \rangle (t)$ where $j < k$. Hence, we can solve (10) for $k= 1$ using the solution for $\langle S_{{0}} \rangle (t)$, then solve (10) for $k= 2$ using the solution for $\langle S_{{0}} \rangle (t)$ or $\langle S_{{1}} \rangle (t)$ (depending on which of these is the parent of node 2), and continue sequentially through all values of $k$.

At each step, we solve (10) using standard methods for first-order constant coefficients linear differential equations, noting that the right hand side will always consist of products and sums of exponential and polynomial functions. Once the functions $\langle S_{{k}} \rangle (t)$ are known, it is straightforward to solve (9b) and (9c) to obtain solutions for $\langle I_{k} \rangle (t)$ and $\langle R_{k} \rangle (t)$.

From these node state probabilities we can obtain the expected number of nodes in any state at any given time by exploiting the fact that the expected value of a sum is the sum of the expected values. Since $\langle S_{{k}} \rangle (t)$ can be thought of as the expected value of an indicator function that is 1 when $X_k= \text {S}$ and 0 otherwise, it follows from the interchangeability of summation and expectation that the expected number of susceptible nodes is given by $\sum _k \langle S_{{k}} \rangle (t)$. This result holds even though node states are not independent and the joint probabilities of node states can be difficult to determine from the node state probabilities.

As a simple example of obtaining closed form solutions for node state probabilities, consider the case where $\lambda _{}$ and $\gamma _{}$ are constant for all nodes. In this case, the symmetry of the system implies that node state probabilities will be identical for nodes of equal depth (*i.e.*, equal distance from the root node). Thus, we can obtain all node state probabilities by considering a simplified problem based on node depths. For clarity of notation, let $d(k)$ represent the depth of node $k$ and let $\bar{s}_{j}$, $\bar{\imath }_{j}$, and $\bar{r}_{j}$ be defined so that $\langle S_{{k}} \rangle = \bar{s}_{d(k)}$ and similarly for $\bar{\imath }_{j}$ and $\bar{r}_{j}$. Note that $d(p(k)) = d(k) - 1$ and that the root node is the only node for which $d(k) = 0$.

Introducing $\bar{s}_{j}$, $\bar{\imath }_{j}$, and $\bar{r}_{j}$, we rearrange (9) and exploit the fact that $\bar{s}_{j} + \bar{\imath }_{j} + \bar{r}_{j} = 1$ to obtain

11a $$\begin{aligned} \frac{\textrm{d} {\bar{s}_{j}}}{\textrm{d} {t}} + (\lambda _{}+\gamma _{}) \bar{s}_{j}&={\left\{ \begin{array}{ll} 0, &{} j= 0, \\ \lambda _{} \bar{s}_{j-1} + \gamma _{}, &{} j\ne 0; \end{array}\right. } \end{aligned}$$

11b $$\begin{aligned} \frac{\textrm{d} {\bar{\imath }_{j}}}{\textrm{d} {t}} + \gamma _{} \bar{\imath }_{j}&= -\frac{\textrm{d} {\bar{s}_{j}}}{\textrm{d} {t}}; \end{aligned}$$

11c $$\begin{aligned} \bar{r}_{j}&= 1 - \bar{s}_{j} - \bar{\imath }_{j}, \end{aligned}$$

which must be solved subject to the initial conditions

12 $$\begin{aligned} \bar{s}_{j}(0)&={\left\{ \begin{array}{ll} 0, &{} j=0, \\ 1, &{} j\ne 0; \end{array}\right. }, \quad \bar{\imath }_{j}(0) ={\left\{ \begin{array}{ll} 1, &{} j=0, \\ 0, &{} j\ne 0. \end{array}\right. } \end{aligned}$$

This system can be solved explicitly using a range of different methods (*e.g.*, operator *D* methods or Laplace transforms). Applying any of these solution methods, we find that

13a $$\begin{aligned} \bar{s}_{j}(t)&= 1 - \frac{\lambda _{}^j}{(\lambda _{} + \gamma _{})^j} + \frac{\lambda _{}^j}{(\lambda _{} + \gamma _{})^j} \textrm{e}^{-(\lambda _{} + \gamma _{})t} \sum _{n=0}^{j-1} \frac{(\lambda _{} + \gamma _{})^nt^n}{n!}, \end{aligned}$$

13b $$\begin{aligned} \bar{\imath }_{j}(t)&= \textrm{e}^{-\gamma _{}t} - \textrm{e}^{-(\lambda _{} + \gamma _{})t} \sum _{n=0}^{j-1} \frac{\lambda _{}^nt^n}{n!}, \end{aligned}$$

13c $$\begin{aligned} \bar{r}_{j}(t)&= \frac{\lambda _{}^j}{(\lambda _{} + \gamma _{})^j} - \textrm{e}^{-\gamma _{}t} + \textrm{e}^{-(\lambda _{} + \gamma _{})t} \sum _{n=0}^{j-1} \left[ \left( \lambda _{}^{n} -\frac{\lambda _{}^j}{(\lambda _{}+\gamma _{})^{j-n}}\right) \frac{t^{n}}{n!} \right] . \end{aligned}$$

To the best of our knowledge, this is the first time that this simple, closed-form solution has been reported in the literature on contagion on networks.

From this solution, it is straightforward to obtain the expected number of nodes in a rooted tree that are susceptible, infectious, or recovered. This can be achieved by counting the number of nodes at each level of the tree and using these counts as weights for a linear combination of the depth-based solutions from (13). For example, if $W_j$ represents the number of nodes at depth *j* from the root, then the expected number of susceptible nodes will be given by $\sum _j W_j \bar{s}_{j}$, where $\bar{s}_{j}$ is taken from (13); equivalent results hold for the expected numbers of infectious and recovered nodes.

**Fig. 1:**
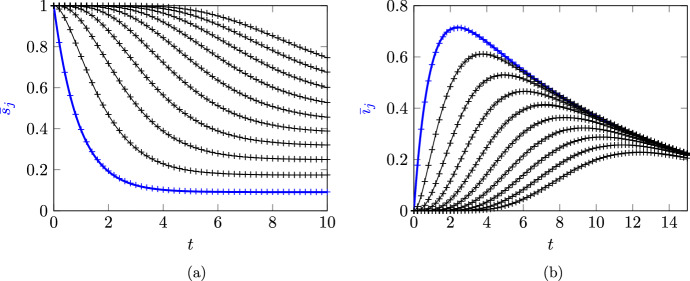
Comparision of the rooted-tree solutions for $\bar{s}_{j}$ and $\bar{\imath }_{j}$ in (13) with simulation results from the average of $10^5$ Gillespie algorithm simulations of the full stochastic SIR model. Subfigure (**a**) shows results for $\bar{s}_{j}$ while subfigure (**b**) shows results for $\bar{\imath }_{j}$ . In both cases, the rooted tree solutions are shown as continuous lines and the numerical results are shown as points marked $+$. Results are shown for nodes where $1 \le d(k) \le 10$; results from $j= d(k) = 1$ are indicated with a thicker blue line and nodes of increasing depthproduce curves further to the right. Parameters used are $\lambda _{} = 1$ and $\gamma _{} = 0.1$

Figure 1 shows comparisons of $\bar{s}_{j}$ and $\bar{\imath }_{j}$ from (13) with empirical node state probabilities based on averaging $10^5$ Gillespie algorithm simulations of the underlying stochastic model. All calculations were performed in Matlab and code is provided at https://github.com/cameronlhall/rootedtreeapprox. These figures illustrate the fact that (13) are exact results; the theoretical results for $\bar{s}_{j}$ and $\bar{\imath }_{j}$ are virtually indistinguishable from results obtained using Gillespie simulations.

Figure 1 also illustrates some properties of SIR dynamics on a chain that can be derived from analysis of (13). For example, (13b) can be rearranged as

14 $$\begin{aligned} \bar{\imath }_{j}(t) = \textrm{e}^{-\gamma _{}t}\left( 1 - \textrm{e}^{-\lambda _{}t} \sum _{n=0}^{j-1} \frac{\lambda _{}^nt^n}{n!} \right) . \end{aligned}$$

Since the sum in (14) is the first *k* terms in the Maclaurin series of $\textrm{e}^{\lambda t}$, we see that $\bar{\imath }_{j}$ will initially be close to zero and will remain close to zero for longer for larger values of $k$. Additionally, we observe that the term in brackets in (14) will asymptotically approach 1 as $t \rightarrow \infty$, which implies that $\bar{\imath }_{j}\sim \textrm{e}^{-\gamma _{}t}$ as $t \rightarrow \infty$. Both the early time behaviour where $\bar{\imath }_{j}$ is close to zero and the late time behaviour where $\bar{\imath }_{j}\sim \textrm{e}^{-\gamma _{}t}$ are visible in Figure 1b.

While (11) and (13) are simple and elegant results, they are of limited practical use because they are specific to rooted trees. Results that only hold on trees are not useful for describing contagion on contact networks or social networks because such networks tend to be highly clustered (Newman 2018) and the clustering coefficient of a tree is necessarily zero.

### Bounds for SIR dynamics on a general network

Despite the limitations of the rooted-tree approximation, a major strength of (11) is that it can be adapted to obtain a node-based approximation of contagion dynamics that gives a bound on $\langle S_{{k}} \rangle$ for all networks. In Sect. 2.2, we showed that the closed system (9) is equivalent to the system (2), which describes the evolution of node state probabilities for SIR dynamics on a rooted tree. In this section, we develop an analogue of (9) that can be applied to a general network. We show that this new formulation yields upper bounds on the functions $\langle S_{{k}} \rangle (t)$.

The formulation developed in this section does not rely on prior knowledge of which neighbour (or neighbours) of a given node might pass the infection to it. Instead, this method constructs a lower bound on the rate that a particular node passes infection to one of its neighbours that is based only on those nodes’ probabilities of being susceptible.

We begin by assuming that no node is recovered at $t = 0$, and so we can specify initial conditions where $\langle S_{{k}} \rangle (0)$ is given for each node and

15 $$\begin{aligned} \langle I_{k} \rangle (0) = 1 - \langle S_{{k}} \rangle (0), \quad \langle R_{k} \rangle (0) = 0. \end{aligned}$$

We make this assumption without loss of generality since the recovered state is permanent in the SIR model; SIR dynamics on a network with initially-recovered nodes will be equivalent to SIR dynamics on a network where those nodes and associated edges have been removed.

The analysis that follows is analogous to the derivation of the exact solution for rooted trees in Section 2.2, but we derive inequalities throughout. Let $j$ and $k$ be chosen so that $j\in {\mathcal {N}}(k)$; that is, the nodes are selected so that it is possible for node $j$ to pass the contagion to node $k$. From the laws of probability, we note that $\langle S_{j} S_{k} \rangle + \langle I_{j} S_{k} \rangle + \langle R_{j} S_{k} \rangle = \langle S_{{k}} \rangle$, and that $\langle S_{j} S_{k} \rangle \le \langle S_{{j}} \rangle$. Combining these gives

16 $$\begin{aligned} \langle I_{j} S_{k} \rangle \ge \langle S_{{k}} \rangle - \langle S_{{j}} \rangle -\langle R_{j} S_{k} \rangle . \end{aligned}$$

Now consider the dynamics of $\langle R_{j} S_{k} \rangle$. We note that a state where $X_{j} = \text {R}$ and $X_{k} = \text {S}$ can only arise from a state where node $X_{j} = \text {I}$ and $X_{k} = \text {S}$. Additionally, a state where $X_{j} = \text {R}$ and $X_{k} = \text {S}$ can change to another state only if node $k$ becomes infected from one of its neighbours. Thus,

17 $$\begin{aligned} \frac{\textrm{d} {\langle R_{j} S_{k} \rangle }}{\textrm{d} {t}} = \gamma _{j} \langle I_{j} S_{k} \rangle - \sum _{i\in {\mathcal {N}}(k)} \lambda _{k \leftarrow i} \langle R_{j} S_{k} I_{i} \rangle , \end{aligned}$$

and, since all probabilities are nonnegative, it follows that

18 $$\begin{aligned} \frac{\textrm{d} {\langle R_{j} S_{k} \rangle }}{\textrm{d} {t}} \le \gamma _{j} \langle I_{j} S_{k} \rangle . \end{aligned}$$

Noting that the terms inside the summation in (1a) are all nonnegative, we observe that

19 $$\begin{aligned} -\frac{\textrm{d} {\langle S_{{k}} \rangle }}{\textrm{d} {t}} \ge \lambda _{k \leftarrow j} \langle I_{j} S_{k} \rangle . \end{aligned}$$

That is, the rate at which node $k$ becomes infectious must be at least as large as the rate at which node $k$ is infected by any particular one of its neighbours, $j$. Combining (18) and (19) then gives

20 $$\begin{aligned} \frac{\textrm{d} {\langle R_{j} S_{k} \rangle }}{\textrm{d} {t}} \le - \frac{\gamma _{j}}{\lambda _{k \leftarrow j}} \frac{\textrm{d} {\langle S_{{k}} \rangle }}{\textrm{d} {t}}. \end{aligned}$$

Using the assumption that no nodes are recovered at $t = 0$, we recall that $\langle R_{j} S_{k} \rangle (0) = 0$. This enables us to integrate (20) from $t = 0$ to obtain $\langle R_{j} S_{k} \rangle (t) \le \frac{\gamma _{j}}{\lambda _{k \leftarrow j}} \left[ \langle S_{{k}} \rangle (0) - \langle S_{{k}} \rangle (t)\right] ,$ and hence (16) becomes

21 $$\begin{aligned} \langle I_{j} S_{k} \rangle \ge \langle S_{{k}} \rangle - \langle S_{{j}} \rangle -\frac{\gamma _{j}}{\lambda _{k \leftarrow j}} \left[ \langle S_{{k}} \rangle (0) - \langle S_{{k}} \rangle (t)\right] . \end{aligned}$$

Thus, we have obtained a lower bound on $\langle I_{j} S_{k} \rangle$ for any pair of neighbouring nodes $j$ and $k$ based only on their probabilities of being susceptible.

In many cases, however, the expression given on the right hand side of (21) will be negative. Since $\langle I_{j} S_{k} \rangle$ must be nonnegative, it follows that

22 $$\begin{aligned} \langle I_{j} S_{k} \rangle (t) \ge \left[ \langle S_{{k}} \rangle (t) -\langle S_{{j}} \rangle (t) - \frac{\gamma _{j}}{\lambda _{k \leftarrow j}} \left[ \langle S_{{k}} \rangle (0) - \langle S_{{k}} \rangle (t)\right] \right] ^{+}, \end{aligned}$$

where $[x]^{+}$ is defined so that

23 $$\begin{aligned} {[}x]^{+} ={\left\{ \begin{array}{ll} 0, &{} x \le 0, \\ x, &{} x > 0. \end{array}\right. } \end{aligned}$$

Substituting into (1a), we obtain

24 $$\begin{aligned} \frac{\textrm{d} {\langle S_{{k}} \rangle }}{\textrm{d} {t}} \le - \sum _{j\in {\mathcal {N}}(k)} \Big [- \gamma _{j} \langle S_{{k}} \rangle (0) + (\lambda _{k \leftarrow j} + \gamma _{j}) \langle S_{{k}} \rangle (t) - \lambda _{k \leftarrow j} \langle S_{{j}} \rangle (t)\Big ]^{+}. \end{aligned}$$

This inequality expresses a lower bound on the rate at which node $k$ can become infected. We note that this lower bound is given as a sum over all neighbouring nodes $j$ of an expression involving only $\langle S_{{k}} \rangle$ and $\langle S_{{j}} \rangle$ (22). For many pairs of neighbouring nodes, this expression will be zero; this does not imply that node $j$ can never infect node $k$ in this case, but instead implies that the method can give no information about the rate at which node $j$ infects $k$.

The differential inequality (24) holds for the true node state probabilities $\langle S_{{k}} \rangle (t)$. Based on this inequality, we now consider the relationship between the true solutions $\langle S_{{k}} \rangle (t)$ and approximate solutions $\langle S^{*}_{{k}} \rangle (t)$ that satisfy the system

25 $$\begin{aligned} \frac{\textrm{d} {\langle S^{*}_{{k}} \rangle }}{\textrm{d} {t}} = - \sum _{j\in {\mathcal {N}}(k)} \Big [- \gamma _{j} \langle S^{*}_{{k}} \rangle (0) + (\lambda _{k \leftarrow j} + \gamma _{j}) \langle S^{*}_{{k}} \rangle (t) - \lambda _{k \leftarrow j} \langle S^{*}_{{j}} \rangle (t)\Big ]^{+}, \end{aligned}$$

subject to initial conditions

26 $$\begin{aligned} \langle S^{*}_{{k}} \rangle (0) = \langle S_{{k}} \rangle (0). \end{aligned}$$

We will show that $\langle S^{*}_{{k}} \rangle (t) \ge \langle S_{{k}} \rangle (t)$ for all $k$ and for all *t*. This follows from the application of Lemma 1 from Simon and Kiss (2018). In order to use this result, we need to show that (25) is a cooperative system of differential equations. This can be done using the Kamke–Müller sufficient conditions (Donnelly 1993; Simon and Kiss 2018), which state that an autonomous system $\frac{\textrm{d} {{\textbf{x}}}}{\textrm{d} {t}} = {\textbf{f}}({\textbf{x}}),$ will be cooperative as long as $f_{k}$ is a nondecreasing function of $x_{j}$ for all $j\ne k$. In our case, we define ${\textbf{x}}$ so that $x_{k} = \langle S^{*}_{{k}} \rangle$, and we define ${\textbf{f}}({\textbf{x}})$ so that

27 $$\begin{aligned} f_{k}({\textbf{x}}) =- \sum _{j\in {\mathcal {N}}(k)} \Big [- \gamma _{j} \langle S^{*}_{{k}} \rangle (0) + (\lambda _{k \leftarrow j} + \gamma _{j}) x_{k} - \lambda _{k \leftarrow j} x_{j}\Big ]^{+}. \end{aligned}$$

Since $f_{k}({\textbf{x}})$ is continuous and the constants $\lambda _{k \leftarrow j}$ are nonnegative, it is clear that $f_{k}$ is a nondecreasing function of $x_{j}$ for all $j$. Hence, the Kamke–Müller conditions are satisfied and (25) is a cooperative system. Using this fact alongside the initial conditions in (26), we apply Lemma 1 from Simon and Kiss (2018) to conclude that $\langle S^{*}_{{k}} \rangle (t) \ge \langle S_{{k}} \rangle (t)$ for all $k$ and for all *t*.

To summarise this result, we can combine (25) with an equation for $\langle I^{*}_{k} \rangle$ based on (1b) to obtain

28a $$\begin{aligned} \frac{\textrm{d} {\langle S^{*}_{{k}} \rangle }}{\textrm{d} {t}}&= - \sum _{j\in {\mathcal {N}}(k)} \Big [- \gamma _{j} \langle S^{*}_{{k}} \rangle (0) + (\lambda _{k \leftarrow j} + \gamma _{j}) \langle S^{*}_{{k}} \rangle (t) - \lambda _{k \leftarrow j} \langle S^{*}_{{j}} \rangle (t)\Big ]^{+}, \end{aligned}$$

28b $$\begin{aligned} \frac{\textrm{d} {\langle I^{*}_{k} \rangle }}{\textrm{d} {t}}&= \sum _{j\in {\mathcal {N}}(k)} \Big [- \gamma _{j} \langle S^{*}_{{k}} \rangle (0) + (\lambda _{k \leftarrow j} + \gamma _{j}) \langle S^{*}_{{k}} \rangle (t) - \lambda _{k \leftarrow j} \langle S^{*}_{{j}} \rangle (t) \Big ]^{+} -\gamma _{k}\langle I^{*}_{k} \rangle (t). \end{aligned}$$

If we also introduce $\langle R^{*}_{k} \rangle = 1 - \langle S^{*}_{{k}} \rangle -\langle I^{*}_{k} \rangle$, this gives a closed system of equations for the approximate dynamics of all node state probabilities. We refer to system (28) as the rooted-tree approximation for SIR dynamics.

If the underlying network is a rooted tree, we can show that (28) is equivalent to (9). To see this, we note that $\langle S^{*}_{{k}} \rangle (t) \le \langle S^{*}_{{k}} \rangle (0)$ for all time and that $\langle S^{*}_{{j}} \rangle (t) \ge \langle S^{*}_{{k}} \rangle (t)$ for any $j\in {\mathcal {N}}(k)$ other than $j= p(k)$. Hence, the terms inside the square brackets in (28) will be nonpositive for any $j\ne p(k)$ and hence applying the positive part operator will yield zero. For a given node $k$ in a rooted tree, the only node $j$ term in (28) that would give a nonzero contribution to the sum will be where $j$ is the parent of $k$.

As a result, the sums in (28) can be simplified in the case of a rooted tree to yield (9). If a network is known to be a rooted tree but the root is not identified, (28) will yield an exact solution without it being necessary to compute the parent of each node, since the parent of each node will be revealed by the only nonzero term in the sums in (28).

**Fig. 2:**
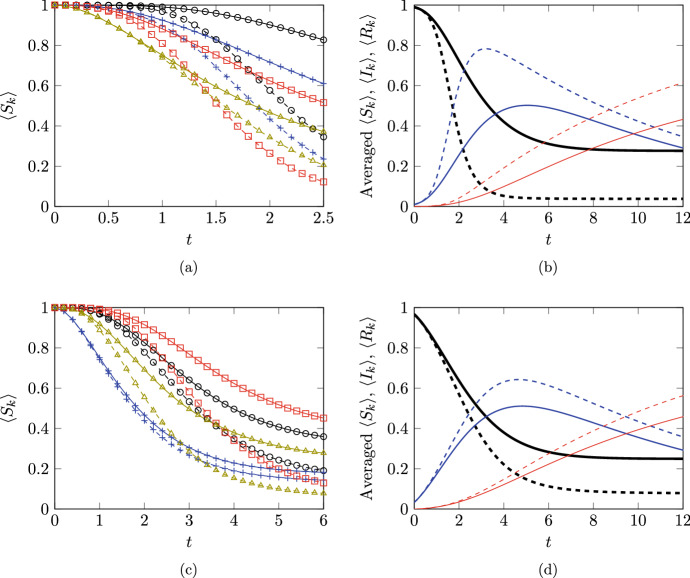
Comparisions of the rooted-tree approximation in (28) with simulation results from the average of $10^5$ Gillespie algorithm simulations of the full stochastic SIR model. Two different networks are illustrated: subfigures (**a**) and (**b**) show results from an Erdős–Rényi random graph of 100 nodes with probability of connection 0.05; subfigures (**c**) and (**d**) show results from a 30-node random tree (generated from a random Prüfer sequence) with 10 additional edges added at random. Subfigures (**a**) and (**c**) show $\langle S_{{k}} \rangle$ for four different nodes: results from the rooted-tree approximation are shown as continuous lines and results from Gillespie simulations are shown as dashed lines; different nodes are distinguished using different colours and marker styles. Subfigures (**b**) and (**d**) show $\langle S_{{k}} \rangle$ (thick black lines), $\langle I_{k} \rangle$ (medium thickness blue lines) and $\langle R_{k} \rangle$ (thin red lines) averaged over all nodes in the network: results from the rooted-tree approximation are shown as continuous lines and results from Gillespie simulations are shown as dashed lines. Parameters used are $\lambda _{} = 1$ and $\gamma _{} = 0.1$. There is a single node that is infectious at $t=0$ and all other nodes are susceptible

Figure 2 shows comparisons of the solution of (28) with results obtained from averaging $10^5$ simulations using the Gillespie algorithm. As previously, Matlab code is available at https://github.com/cameronlhall/rootedtreeapprox. Two different networks are shown: an Erdős–Rényi (ER) random graph (Fig.  2a and b) and a network that is ‘almost’ a tree (Fig. 2c and d) in the sense that it was constructed from a random tree by adding some additional edges at random.

If we think of the Gillespie algorithm results as being the ‘true’ solution, we see from Fig. 2a and c that the rooted-tree approximation does indeed give an upper bound on $\langle S_{{k}} \rangle$ for each individual node $k$. Throughout Fig. 2a and c we see that the rooted-tree approximation deviates from the true solutions by different amounts at different times for different nodes, but the difference is typically substantial as time goes on. This deviation is observed for the ‘almost tree’ in Fig. 2c as well as for the ER graph in Fig. 2a, although we note that the difference between the approximation and the true solution grows faster and becomes larger in the case of the ER graph.

The overall differences between the rooted-tree approximation and the true solution are best seen in Fig. 2b and d. These show $\langle S_{{k}} \rangle (t)$, $\langle I_{k} \rangle (t)$ and $\langle R_{k} \rangle (t)$ averaged over all nodes in the network. As may be anticipated from Fig. 2a and c, the rooted-tree approximation gives a overestimate of $\langle S_{{k}} \rangle$ (including the equilibrium $\langle S_{{k}} \rangle$ as $t \rightarrow \infty$) and underestimates the peak in $\langle I_{k} \rangle$. Overall, we see that (28) does indeed give bounds on $\langle S_{{k}} \rangle$ but that these bounds are not generally very tight.

## Rooted-tree approximation for a generalised SEIR model

### Preliminaries

The Susceptible-Exposed-Infectious-Recovered (SEIR) model is a well-established compartment model in the epidemiological literature (Brauer et al. 2019). The SEIR model differs from the SIR model by the introduction of an ‘exposed’ or ‘latent’ state representing individuals that have encountered the disease but are not yet infectious. Some SEIR models involve multiple classes of exposed state; such models have been analysed mathematically (Bame et al. 2008; Diekmann et al. 2010; Guo et al. 2012) and applied to modelling certain diseases (Cunniffe et al. 2012).

As with the SIR model, the SEIR model has also been extended to networks (Kang et al. 2020; Liu et al. 2017; Newman 2018; Pastor-Satorras et al. 2015). For the most part, network SEIR models in the published literature involve a single exposed state; however, they can be extended to multiple classes of exposed state in an analogous way to compartment models. Our analysis of SIR models in Sect. 2 can be extended to SEIR models, including in a general setting with arbitrarily many distinct exposed states. In this section, we replicate our analysis from the previous section but for generalised SEIR models: we construct a node-based approximation of SEIR contagion dynamics that is exact on rooted trees and that yields an upper bound on $\langle S_{{k}} \rangle (t)$ on more general graphs.

In our generalised network SEIR model, each node represents an individual, so that at any time a node can either be susceptible (S), exposed of class $u$ (E$^{(u)}$), infectious (I), or recovered (R). We assume that there are finitely many ($N_u$) different classes of exposed nodes. Susceptible nodes in contact with infectious nodes may become exposed (in any class) or infectious; we refer to the process of a susceptible node changing its state as ‘infection’ regardless of whether the node becomes exposed or infectious. Exposed nodes may change to a different class of exposed, become infectious, or recover; we assume that exposed nodes cannot become susceptible. Infectious nodes may recover, but cannot become exposed or susceptible. Once a node has recovered, it remains recovered for all time.

Each of these transitions is governed by a different rate parameter. The rate of infection (*i.e.*, the total rate at which a susceptible node in contact with an infectious node becomes exposed or infectious) is given by $\lambda _{}$. The probability that a susceptible node becomes exposed of class $u$ when infection occurs is given by ${\varphi }^{(u)}_{}$; hence, the probability that a susceptible node becomes infectious when infection occurs is $1 - \sum _{u} {\varphi }^{(u)}_{}$. The rate at which an exposed node of class $u$ becomes an exposed node of class $v$ is given by $b^{(v \leftarrow u)}_{}$. The rate at which an exposed node of class $u$ becomes infectious is given by $\mu ^{(u)}_{}$. The rate at which an exposed node of class $u$ recovers is given by $\nu ^{(u)}_{}$. The rate at which an infectious node recovers is given by $\gamma _{}$. These different transitions are summarised below:

$$\begin{aligned} \text {S (with I)}&\xrightarrow [\qquad \qquad \quad ]{\lambda _{} {\varphi }^{(u)}_{}} \text {E}^{(u)}\quad \text {E}^{(u)} \xrightarrow [\qquad \qquad \quad ]{b^{(v \leftarrow u)}_{}} \text {E}^{(v)} \quad \text {E}^{(u)} \xrightarrow [\qquad \qquad \quad ]{\nu ^{(u)}_{}} \text {R} \\ \text {S (with I)}&\xrightarrow [\qquad \qquad \quad ]{\lambda _{} (1 - \sum {\varphi }^{(u)}_{})} \text {I} \qquad ~\text {E}^{(u)} \xrightarrow [\qquad \qquad \quad ]{\mu ^{(u)}_{}} \text {I} \qquad \,\,\quad ~~\text {I} \xrightarrow [\qquad \qquad \quad ]{\gamma _{}} \text {R} \end{aligned}$$

As in Sect. 2.1, we assume that the model parameters can depend on the relevant edge or node, and we represent this using subscripts. The most general approach would be to permit both $\lambda _{}$ and ${\varphi }^{(u)}_{}$ to be edge-dependent; however, this level of generality in ${\varphi }^{(u)}_{}$ would lead to a problem with the bounding argument in Sect. 3.3. To circumvent this, we permit ${\varphi }^{(u)}_{}$ to depend on the recipient node but not on the infecting node; that is, we assume ${\varphi }^{(u)}_{k \leftarrow j} = {\varphi }^{(u)}_{k}$. Physically, this would correspond to a situation where individual responses to infection (*e.g.*, whether an individual immediately becomes infectious or whether they first enter an exposed state) may vary between individuals but do not depend on the source of infection.

To assist with the analysis of the $N_u$ different classes of exposed state, we introduce the $N_u$-dimensional vectors $\langle {\textbf{E}}_{k} \rangle (t)$, $\varvec{\varphi }_{k}$, $\varvec{\nu }_{k}$, $\varvec{\mu }_{k}$, ${\textbf{e}}$, and $\varvec{0}$ so that

29 $$\begin{aligned} \langle {\textbf{E}}_{k} \rangle (t)&=\begin{bmatrix} \langle E_{k}^{(1)} \rangle (t) \\ \langle E_{k}^{(2)} \rangle (t) \\ \vdots \\ \langle E_{k}^{(N_u)} \rangle (t) \end{bmatrix}, \quad \varvec{\varphi }_{k} = \begin{bmatrix} {\varphi }^{(1)}_{k} \\ {\varphi }^{(2)}_{k} \\ \vdots \\ {\varphi }^{(N_u)}_{k} \end{bmatrix}, \quad \varvec{\nu }_{k}= \begin{bmatrix} \nu ^{(1)}_{k} \\ \nu ^{(2)}_{k} \\ \vdots \\ \nu ^{(N_u)}_{k} \end{bmatrix}, \end{aligned}$$

30 $$\begin{aligned} \varvec{\mu }_{k}&= \begin{bmatrix} \mu ^{(1)}_{k} \\ \mu ^{(2)}_{k} \\ \vdots \\ \mu ^{(N_u)}_{k} \end{bmatrix}, \quad {\textbf{e}}= \begin{bmatrix} 1 \\ 1 \\ \vdots \\ 1 \end{bmatrix}, \quad \varvec{0}= \begin{bmatrix} 0 \\ 0 \\ \vdots \\ 0 \end{bmatrix}. \end{aligned}$$

We note that $0 \le {\textbf{e}}\cdot \varvec{\varphi }_{k} \le 1$ for all $k$, and that the rate at which a susceptible node $k$ in contact with an infectious node $j$ becomes infectious is given by

31 $$\begin{aligned} \lambda _{k \leftarrow j} \left( 1 - \sum _{u=1}^{N_u} {\varphi }^{(u)}_{k}\right) = \lambda _{k \leftarrow j} \left( 1 - {\textbf{e}}\cdot \varvec{\varphi }_{k} \right) . \end{aligned}$$

Lastly, we define the $N_u$-by-$N_u$ matrix ${\textbf{B}}_{k}$ so that

32 $$\begin{aligned} \left[ {\textbf{B}}_{k}\right] _{uv} ={\left\{ \begin{array}{ll} \mu ^{(v)}_{k} + \nu ^{(v)}_{k} + \displaystyle \sum _{\begin{array}{c} w = 1 \\ w \ne v \end{array}}^{N_u} b^{(w \leftarrow v)}_{k}, &{} u= v, \\ -b^{(u \leftarrow v)}_{k}; &{} u\ne v. \end{array}\right. } \end{aligned}$$

With this notation, the dynamics of contagion on any network can be described using the following equations:

33a $$\begin{aligned} \frac{\textrm{d} {\langle S_{{k}} \rangle }}{\textrm{d} {t}}&= - \sum _{j \in {\mathcal {N}}(k)} \lambda _{k \leftarrow j} \langle I_{j} S_{k} \rangle , \end{aligned}$$

33b $$\begin{aligned} \frac{\textrm{d} {\langle {\textbf{E}}_{k} \rangle }}{\textrm{d} {t}}&= \varvec{\varphi }_{k} \sum _{j \in {\mathcal {N}}(k)} \lambda _{k \leftarrow j} \langle I_{j} S_{k} \rangle - {\textbf{B}}_{k} \langle {\textbf{E}}_{k} \rangle , \end{aligned}$$

33c $$\begin{aligned} \frac{\textrm{d} {\langle I_{k} \rangle }}{\textrm{d} {t}}&= (1 - {\textbf{e}}\cdot \varvec{\varphi }_{k}) \sum _{j \in {\mathcal {N}}(k)} \lambda _{k \leftarrow j} \langle I_{j} S_{k} \rangle + \varvec{\mu }_{k} \cdot \langle {\textbf{E}}_{k} \rangle - \gamma _{k} \langle I_{k} \rangle , \end{aligned}$$

33d $$\begin{aligned} \frac{\textrm{d} {\langle R_{k} \rangle }}{\textrm{d} {t}}&= \varvec{\nu }_{k} \cdot \langle {\textbf{E}}_{k} \rangle +\gamma _{k} \langle I_{k} \rangle , \end{aligned}$$

which must be solved subject to suitable initial conditions.

Note that if $\varvec{\varphi }_{}$ were permitted to depend on the source of infection as well as on the node that becomes infected then the corresponding $\varvec{\varphi }_{k\leftarrow j}$ terms would need to be included inside the summations in equations (33b) and (33c).

Note also that (33a) can be used to express (33b) and (33c) in the equivalent forms

34b $$\begin{aligned} \frac{\textrm{d} {\langle {\textbf{E}}_{k} \rangle }}{\textrm{d} {t}}&= -\varvec{\varphi }_{k} \frac{\textrm{d} {\langle S_{{k}} \rangle }}{\textrm{d} {t}} - {\textbf{B}}_{k} \langle {\textbf{E}}_{k} \rangle , \end{aligned}$$

34c $$\begin{aligned} \frac{\textrm{d} {\langle I_{k} \rangle }}{\textrm{d} {t}}&= -(1 - {\textbf{e}}\cdot \varvec{\varphi }_{k}) \frac{\textrm{d} {\langle S_{{k}} \rangle }}{\textrm{d} {t}} + \varvec{\mu }_{k} \cdot \langle {\textbf{E}}_{k} \rangle -\gamma _{k} \langle I_{k} \rangle . \end{aligned}$$

Given the length of the expressions that we obtain for $\lambda _{k \leftarrow j} \langle I_{j} S_{k} \rangle$ in our analysis, we will sometimes prefer (34b) and (34c) over (33b) and (33c) for concision.

### Exact SEIR dynamics on a rooted tree

As in Sect. 2.2, we begin by considering contagion dynamics on a rooted tree, where there is a single node, $k = 0$, which is the source of infection. This node may either be exposed or infectious at $t = 0$. Introducing equivalent notation and following the same logic as for the derivation of (2), we find that the evolution equations for node state probabilities on a rooted tree are

35a $$\begin{aligned} \frac{\textrm{d} {\langle S_{{k}} \rangle }}{\textrm{d} {t}}&={\left\{ \begin{array}{ll} 0, &{} k= 0, \\ -\lambda _{k} \langle I_{p(k)} S_{k} \rangle , &{} k\ne 0; \end{array}\right. } \end{aligned}$$

35b $$\begin{aligned} \frac{\textrm{d} {\langle {\textbf{E}}_{k} \rangle }}{\textrm{d} {t}}&={\left\{ \begin{array}{ll} - {\textbf{B}}_{k} \langle {\textbf{E}}_{k} \rangle , &{} k= 0, \\ \lambda _{k} \varvec{\varphi }_{k} \langle I_{p(k)} S_{k} \rangle - {\textbf{B}}_{k} \langle {\textbf{E}}_{k} \rangle , &{} k\ne 0; \end{array}\right. } \end{aligned}$$

35c $$\begin{aligned} \frac{\textrm{d} {\langle I_{k} \rangle }}{\textrm{d} {t}}&={\left\{ \begin{array}{ll} \varvec{\mu }_{k} \cdot \langle {\textbf{E}}_{k} \rangle - \gamma _{k} \langle I_{k} \rangle , &{} k= 0, \\ \lambda _{k}(1 - {\textbf{e}}\cdot \varvec{\varphi }_{k}) \langle I_{p(k)} S_{k} \rangle + \varvec{\mu }_{k} \cdot \langle {\textbf{E}}_{k} \rangle - \gamma _{k} \langle I_{k} \rangle , &{} k\ne 0 ; \end{array}\right. } \end{aligned}$$

35d $$\begin{aligned} \frac{\textrm{d} {\langle R_{k} \rangle }}{\textrm{d} {t}}&= \varvec{\nu }_{k} \cdot \langle {\textbf{E}}_{k} \rangle + \gamma _{k} \langle I_{k} \rangle . \end{aligned}$$

These equations need to be solved subject to initial conditions where

36 $$\begin{aligned} \langle S_{{k}} \rangle (0)&= 1, \quad \langle {\textbf{E}}_{k} \rangle (0) = \varvec{0}, \quad \langle I_{k} \rangle (0) = \langle R_{k} \rangle (0) = 0, \quad k\ne 0, \end{aligned}$$

and where $\langle {\textbf{E}}_{0} \rangle (0) = \langle {\textbf{E}}_0 \rangle ^{\textrm{init}}$ and $\langle I_{0} \rangle (0) = \langle I_0 \rangle ^{\textrm{init}}$ are specified, but $\langle S_{{0}} \rangle (0) = \langle R_{0} \rangle (0) = 0$. We note that $\varvec{\varphi }_{0}$ does not appear in system (35) or in the initial conditions. As we will see, it will be convenient to define $\varvec{\varphi }_{0}$ so that $\varvec{\varphi }_{0} = \langle {\textbf{E}}_0 \rangle ^{\textrm{init}}$, and hence $\langle I_0 \rangle ^{\textrm{init}}= 1 - {\textbf{e}}\cdot \varvec{\varphi }_{0}$.

System (35) is not closed because of the presence of $\langle I_{p(k)} S_{k} \rangle$. As in Sect. 2.2, we exploit the properties of a rooted tree to find an expression for $\langle I_{p(k)} S_{k} \rangle$ in terms of the node state probabilities and hence obtain a closed system. Since the parent node of node 0 is not defined, we assume (unless otherwise specified) that $k\ne 0$ in all analysis below where $p(k)$ is mentioned.

We begin by noting that the law of total probability gives

37 $$\begin{aligned} \langle S_{{k}} \rangle = \langle S_{p(k)} S_{k} \rangle +\sum _{u=1}^{N_u} \langle E_{p(k)}^{(u)} S_{k} \rangle +\langle I_{p(k)} S_{k} \rangle + \langle R_{p(k)} S_{k} \rangle . \end{aligned}$$

The fact that infection can only spread from node $p(k)$ to node $k$ and not *vice versa* means that if either $X_{p(k)} = \text {S}$ or $X_{p(k)} = \text {E}^{(j)}$ then $X_{k} = \text {S}$. Hence, $\langle S_{p(k)} S_{k} \rangle = \langle S_{{p(k)}} \rangle$ and $\langle E_{p(k)}^{(u)} S_{k} \rangle = \langle E_{p(k)}^{(u)} \rangle$. Thus, (37) can be rearranged to give

38 $$\begin{aligned} \langle I_{p(k)} S_{k} \rangle (t)&= \langle S_{{k}} \rangle -\langle S_{{p(k)}} \rangle (t) - \sum _{u=1}^{N_u} \langle E_{p(k)}^{(u)} \rangle - \langle R_{p(k)} S_{k} \rangle \nonumber \\&= \langle S_{{k}} \rangle - \langle S_{{p(k)}} \rangle - {\textbf{e}}\cdot \langle {\textbf{E}}_{p(k)} \rangle - \langle R_{p(k)} S_{k} \rangle . \end{aligned}$$

As previously, we now seek a differential equation for $\langle R_{p(k)} S_{k} \rangle$ that can be directly integrated to obtain $\langle R_{p(k)} S_{k} \rangle$ in terms of node state probabilities. The only way to achieve a state where $X_{p(k)} = \text {R}$ and $X_{k} = \text {S}$ is for node $p(k)$ to recover (either form an exposed state or an infectious state) while node $k$ is susceptible. Once node $p(k)$ has recovered, this state will then be permanent. Since $\langle E_{p(k)}^{(u)} S_{k} \rangle = \langle E_{p(k)}^{(u)} \rangle$, it therefore follows that

39 $$\begin{aligned} \frac{\textrm{d} {\langle R_{p(k)} S_{k} \rangle }}{\textrm{d} {t}} =\varvec{\nu }_{p(k)} \cdot \langle {\textbf{E}}_{p(k)} \rangle +\gamma _{p(k)} \langle I_{p(k)} S_{k} \rangle . \end{aligned}$$

Using (35a), this rearranges to give

40 $$\begin{aligned} \frac{\textrm{d} {\langle R_{p(k)} S_{k} \rangle }}{\textrm{d} {t}} =\varvec{\nu }_{p(k)} \cdot \langle {\textbf{E}}_{p(k)} \rangle -\frac{\gamma _{p(k)}}{\lambda _{k}} \frac{\textrm{d} {\langle S_{{k}} \rangle }}{\textrm{d} {t}}, \end{aligned}$$

The next step is to rewrite $\varvec{\nu }_{p(k)} \cdot \langle {\textbf{E}}_{p(k)} \rangle (t)$ in terms of the derivatives of node state probabilities. For any node $k$ (including $k= 0$), let ${\textbf{M}}_{k}$ be the block matrix defined by

41 $$\begin{aligned} {\textbf{M}}_{k} =\begin{bmatrix} 1 &{} \varvec{0}^T \\ - \varvec{\varphi }_{k} &{} {\textbf{B}}_{k} \end{bmatrix}, \end{aligned}$$

so that the block matrix inversion formula (Petersen and Pedersen 2012) gives

42 $$\begin{aligned} {\textbf{M}}_{k}^{-1} =\begin{bmatrix} 1 &{} \varvec{0}^T \\ {\textbf{B}}_{k}^{-1} \varvec{\varphi }_{k} &{} {\textbf{B}}_{k}^{-1} \end{bmatrix}. \end{aligned}$$

Using ${\textbf{M}}_{k}$, we can rewrite equations (35a) and (35b) together as

43 $$\begin{aligned} \begin{bmatrix} \frac{\textrm{d} {\langle S_{{k}} \rangle }}{\textrm{d} {t}} \\ \frac{\textrm{d} {\langle {\textbf{E}}_{k} \rangle }}{\textrm{d} {t}} \end{bmatrix} =- {\textbf{M}}_{k}\begin{bmatrix} \lambda _{k} \langle I_{p(k)} S_{k} \rangle \\ \langle {\textbf{E}}_{k} \rangle \end{bmatrix}. \end{aligned}$$

If we assert that $\langle I_{p(0)} S_{0} \rangle (t) \equiv 0$, then (43) also applies when $k= 0$.

We now use ${\textbf{M}}_{p(k)}$ to express $\varvec{\nu }_{p(k)} \cdot \langle {\textbf{E}}_{p(k)} \rangle$ in terms of derivatives as follows:

44 $$\begin{aligned} \varvec{\nu }_{p(k)} \cdot \langle {\textbf{E}}_{p(k)} \rangle&=\begin{bmatrix} 0&\varvec{\nu }_{p(k)}^T \end{bmatrix} \begin{bmatrix} \lambda _{p(k)}\langle I_{p[p(k)]} S_{p(k)} \rangle \\ \langle {\textbf{E}}_{p(k)} \rangle \end{bmatrix} \end{aligned}$$

45 $$\begin{aligned}&=- \begin{bmatrix} 0&\varvec{\nu }_{p(k)}^T \end{bmatrix} {\textbf{M}}_{p(k)}^{-1} \begin{bmatrix} \frac{\textrm{d} {\langle S_{{p(k)}} \rangle }}{\textrm{d} {t}} \\ \frac{\textrm{d} {\langle {\textbf{E}}_{p(k)} \rangle }}{\textrm{d} {t}} \end{bmatrix} \nonumber \\&= -\varvec{\nu }_{p(k)}^T {\textbf{B}}_{p(k)}^{-1} \varvec{\varphi }_{p(k)} \frac{\textrm{d} {\langle S_{{p(k)}} \rangle }}{\textrm{d} {t}} - \varvec{\nu }_{p(k)}^T {\textbf{B}}_{p(k)}^{-1} \frac{\textrm{d} {\langle {\textbf{E}}_{p(k)} \rangle }}{\textrm{d} {t}}. \end{aligned}$$

Note that equation (45) applies even when $p(k) = 0$; even though the value of $\langle I_{p[p(k)]} S_{p(k)} \rangle$ would be undefined in (44), it is multiplied by zero and does not affect the final result.

Substituting (45) into (40) yields

46 $$\begin{aligned} \frac{\textrm{d} {\langle R_{p(k)} S_{k} \rangle }}{\textrm{d} {t}} =-\varvec{\nu }_{p(k)}^T {\textbf{B}}_{p(k)}^{-1} \varvec{\varphi }_{p(k)} \frac{\textrm{d} {\langle S_{{p(k)}} \rangle }}{\textrm{d} {t}} -\varvec{\nu }_{p(k)}^T {\textbf{B}}_{p(k)}^{-1} \frac{\textrm{d} {\langle {\textbf{E}}_{p(k)} \rangle }}{\textrm{d} {t}} -\frac{\gamma _{p(k)}}{\lambda _{k}} \frac{\textrm{d} {\langle S_{{k}} \rangle }}{\textrm{d} {t}}, \end{aligned}$$

and hence we find that

47 $$\begin{aligned} \langle R_{p(k)} S_{k} \rangle = C_{k} -\varvec{\nu }_{p(k)}^T {\textbf{B}}_{p(k)}^{-1} \varvec{\varphi }_{p(k)} \langle S_{{p(k)}} \rangle -\varvec{\nu }_{p(k)}^T {\textbf{B}}_{p(k)}^{-1} \langle {\textbf{E}}_{p(k)} \rangle - \frac{\gamma _{p(k)}}{\lambda _{k}} \langle S_{{k}} \rangle , \end{aligned}$$

where $C_{k}$ is a constant to be determined from the initial conditions.

In the case where $p(k) \ne 0$, the initial conditions in (36) yield

48 $$\begin{aligned} C_{k} = \varvec{\nu }_{p(k)}^T {\textbf{B}}_{p(k)}^{-1} \varvec{\varphi }_{p(k)} + \frac{\gamma _{p(k)}}{\lambda _{k}}. \end{aligned}$$

In the case where $p(k) = 0$, the initial conditions yield

49 $$\begin{aligned} C_{k} = \varvec{\nu }_{0}^T {\textbf{B}}_{0}^{-1} \langle {\textbf{E}}_0 \rangle ^{\textrm{init}}+\frac{\gamma _{0}}{\lambda _{k}}. \end{aligned}$$

As noted previously, this motivates us to define $\varvec{\varphi }_{0} = \langle {\textbf{E}}_0 \rangle ^{\textrm{init}}$ so that (48) can be used to give the constant $C_{k}$ for all nodes $k\ne 0$.

Combining (47) and (48), we obtain an expression for $\langle R_{p(k)} S_{k} \rangle$ that can be substituted into (38) to yield

50 $$\begin{aligned} \langle I_{p(k)} S_{k} \rangle&=- \varvec{\nu }_{p(k)}^T{\textbf{B}}_{p(k)}^{-1} \varvec{\varphi }_{p(k)} - \frac{\gamma _{p(k)}}{\lambda _{k}} + \frac{\lambda _{k} + \gamma _{p(k)}}{\lambda _{k}}\langle S_{{k}} \rangle \nonumber \\&\quad - \left( 1 - \varvec{\nu }_{p(k)}^{T} {\textbf{B}}_{p(k)}^{-1} \varvec{\varphi }_{p(k)} \right) \langle S_{{p(k)}} \rangle -\left( {\textbf{e}}- {\textbf{B}}_{p(k)}^{-T} \varvec{\nu }_{p(k)} \right) \cdot \langle {\textbf{E}}_{p(k)} \rangle . \end{aligned}$$

We note that (32) implies that

51 $$\begin{aligned} \sum _{u=1}^{N_u} \left[ {\textbf{B}}_{k}\right] _{uv} =\mu ^{(v)}_{k} + \nu ^{(v)}_{k}, \end{aligned}$$

and hence ${\textbf{B}}_{k}^T {\textbf{e}}= \varvec{\mu }_{k} + \varvec{\nu }_{k}$. This rearranges to yield ${\textbf{B}}_{k}^{-T} \varvec{\mu }_{k} = {\textbf{e}}- {\textbf{B}}_{k}^{-T} \varvec{\nu }_{k}$ so that (50) becomes

52 $$\begin{aligned} \langle I_{p(k)} S_{k} \rangle&=- \varvec{\nu }_{p(k)}^T{\textbf{B}}_{p(k)}^{-1} \varvec{\varphi }_{p(k)} - \frac{\gamma _{p(k)}}{\lambda _{k}} + \frac{\lambda _{k} + \gamma _{p(k)}}{\lambda _{k}}\langle S_{{k}} \rangle \nonumber \\&\quad - \left( 1 - \varvec{\nu }_{p(k)}^{T} {\textbf{B}}_{p(k)}^{-1} \varvec{\varphi }_{p(k)} \right) \langle S_{{p(k)}} \rangle - \varvec{\mu }_{p(k)}^T{\textbf{B}}_{p(k)}^{-1} \langle {\textbf{E}}_{p(k)} \rangle . \end{aligned}$$

As an aside, we note from (32) that ${\textbf{B}}_{k}^T$ is a strictly diagonally dominant matrix with positive diagonal entries. From Berman and Plemmons (1994), it follows that ${\textbf{B}}_{k}^T$ is inverse-positive. Hence, the elements of ${\textbf{B}}_{k}^{-T} \varvec{\mu }_{k}$ and ${\textbf{B}}_{k}^{-T} \varvec{\nu }_{k}$ are all between 0 and 1 (inclusive) and we note that the coefficients of $\langle S_{{p(k)}} \rangle$ and $\langle E_{p(k)}^{(u)} \rangle$ in (52) are all nonpositive.

Using (52) and (34), system (35) can be rearranged to give

53a $$\begin{aligned} \frac{\textrm{d} {\langle S_{{k}} \rangle }}{\textrm{d} {t}}&=0, \quad k=0, \end{aligned}$$

53b $$\begin{aligned} \frac{\textrm{d} {\langle S_{{k}} \rangle }}{\textrm{d} {t}}&= - \left( \lambda _{k} + \gamma _{p(k)}\right) \langle S_{{k}} \rangle +\lambda _{k} \left( 1 - \varvec{\nu }_{p(k)}^{T} {\textbf{B}}_{p(k)}^{-1} \varvec{\varphi }_{p(k)} \right) \langle S_{{p(k)}} \rangle \nonumber \\&\qquad + \lambda _{k} \varvec{\mu }_{p(k)}^T{\textbf{B}}_{p(k)}^{-1} \langle {\textbf{E}}_{p(k)} \rangle + \gamma _{p(k)} - \lambda _{k} \varvec{\nu }_{p(k)}^T{\textbf{B}}_{p(k)}^{-1} \varvec{\varphi }_{p(k)}, \quad k\ne 0, \end{aligned}$$

53c $$\begin{aligned} \frac{\textrm{d} {\langle {\textbf{E}}_{k} \rangle }}{\textrm{d} {t}}&= -\varvec{\varphi }_{k} \frac{\textrm{d} {\langle S_{{k}} \rangle }}{\textrm{d} {t}} - {\textbf{B}}_{k} \langle {\textbf{E}}_{k} \rangle , \end{aligned}$$

53d $$\begin{aligned} \frac{\textrm{d} {\langle I_{k} \rangle }}{\textrm{d} {t}}&= -(1 - {\textbf{e}}\cdot \varvec{\varphi }_{k}) \frac{\textrm{d} {\langle S_{{k}} \rangle }}{\textrm{d} {t}} - \gamma _{k} \langle I_{k} \rangle +\varvec{\mu }_{k} \cdot \langle {\textbf{E}}_{k} \rangle , \end{aligned}$$

53e $$\begin{aligned} \frac{\textrm{d} {\langle R_{k} \rangle }}{\textrm{d} {t}}&= \varvec{\nu }_{k} \cdot \langle {\textbf{E}}_{k} \rangle +\gamma _{k} \langle I_{k} \rangle . \end{aligned}$$

As for the SIR model in Sect. 2.2, this is a partially-decoupled system. To see this, we observe that the dynamics of $\langle S_{{k}} \rangle$ in (53b) are independent of $\langle {\textbf{E}}_{k} \rangle$; instead, $\frac{\textrm{d} {\langle S_{{k}} \rangle }}{\textrm{d} {t}}$ depends only on $\langle S_{{k}} \rangle$ and the node state probabilities at the parent node. Since equations (53b) and (53c) are both independent of $\langle I_{k} \rangle (t)$ and $\langle R_{k} \rangle (t)$, this implies that (53) can be solved from the root outwards, with $\langle S_{{k}} \rangle$ solved before $\langle {\textbf{E}}_{k} \rangle$ at each subsequent node.

Moreover, consider the case where exposed states are traversed in order—that is, where $b^{(u \leftarrow v)}_{k}$ is zero whenever $u< v$). This situation is physically plausible, since it corresponds to a case where a diseased individual can progress through different exposed “stages” before becoming infectious or recovering, but can never return to an earlier class of exposed state from a more advanced class. In this case, the matrix ${\textbf{B}}_{k}$ will be lower triangular and hence the scalar equations that constitute (53c) will also be partially decoupled. Since system (53) is linear, this implies that the full solution can be obtained exactly by the sequential solving of linear scalar ordinary differential equations; it is not even necessary to solve an eigenvalue problem in order to obtain the exact solution to SEIR dynamics on a rooted tree. While we do not present closed-form solutions here, it is theoretically possible to obtain results analogous to (53) using standard methods for nonhomogeneous constant-coefficients differential equations.

As in Sect. 2.3, we test the rooted-tree formulation in system (53) by considering SEIR dynamicson a chain. For simplicity, we consider the case where there is a single class of exposed state and so the vectors and matrices in (53) can be replaced by scalars. Noting that the equivalent of ${\textbf{B}}_{k}$ will be ${\mu }_{k} + {\nu }_{k}$, this leads to the system

54a $$\begin{aligned} \frac{\textrm{d} {\langle S_{{k}} \rangle }}{\textrm{d} {t}}&=0, \quad k=0, \end{aligned}$$

54b $$\begin{aligned} \frac{\textrm{d} {\langle S_{{k}} \rangle }}{\textrm{d} {t}}&= \frac{\lambda _{k} {\mu }_{k-1} }{{\mu }_{k-1} + {\nu }_{k-1}} \left( {\varphi }_{k-1}\langle S_{{k-1}} \rangle + \langle E_{k-1} \rangle \right) \nonumber \\&\qquad - \left( \lambda _{k} + \gamma _{k-1}\right) \langle S_{{k}} \rangle + \gamma _{k-1} - \frac{\lambda _{k} {\nu }_{k-1} {\varphi }_{k-1} }{{\mu }_{k-1} + {\nu }_{k-1}}, \quad k\ne 0, \end{aligned}$$

54c $$\begin{aligned} \frac{\textrm{d} {\langle E_{k} \rangle }}{\textrm{d} {t}}&= -{\varphi }_{k} \frac{\textrm{d} {\langle S_{{k}} \rangle }}{\textrm{d} {t}} - ({\mu }_{k} + {\nu }_{k}) \langle E_{k} \rangle , \end{aligned}$$

54d $$\begin{aligned} \frac{\textrm{d} {\langle I_{k} \rangle }}{\textrm{d} {t}}&= -(1 - {\varphi }_{k}) \frac{\textrm{d} {\langle S_{{k}} \rangle }}{\textrm{d} {t}} - \gamma _{k} \langle I_{k} \rangle +{\mu }_{k} \langle E_{k} \rangle , \end{aligned}$$

**Fig. 3:**
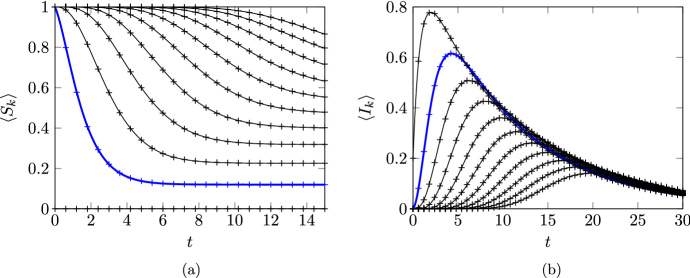
Comparision of the rooted-tree solutions for $\langle S_{{k}} \rangle$ and $\langle I_{k} \rangle$ based on numerical solution of (54) with simulation results from the average of $10^5$ Gillespie algorithm simulations of the full stochastic model. Subfigure (**a**) shows results for $\langle S_{{k}} \rangle$ while subfigure (**b**) shows results for $\langle I_{k} \rangle$. In both cases, the rooted tree solutions are shown as continuous lines and the numerical results are shown as points marked $+$. Results are shown for the first eleven nodes (from $k= 0$ to $k= 10$); results from $k= 1$ are indicated with a thicker blue line and subsequent nodes produce curves further to the right. Parameters used are $\lambda _{} = 1$, ${\varphi }_{} = 0.8$, ${\mu }_{} = 1.2$, ${\nu }_{} = 0.05$, and $\gamma _{} = 0.1$. For consistency with the value of ${\varphi }_{}$, the initial conditions are $\langle I_0 \rangle ^{\textrm{init}}= 0.2$ and $\langle E_0 \rangle ^{\textrm{init}}= 0.8$

Figure 3 shows a comparison of $\langle S_{{k}} \rangle (t)$ and $\langle I_{k} \rangle (t)$ obtained from the numerical solution of (54) with the average of $10^5$ Gillespie algorithm simulations of the underlying stochastic model (code again available at https://github.com/cameronlhall/rootedtreeapprox). As in Fig. 1, this exemplifies the fact that system (54) is exact; the two sets of results are virtually indistinguishable.

### Bounds for SEIR dynamics on a general network

We now replicate the argument in Sect. 2.4 to obtain bounds on the solution of generalised SEIR dynamics on a general network. In this case our starting point is system (33) and we assume without loss of generality that $\langle R_{k} \rangle (0) = 0$ for all nodes.

By analogous arguments to Sect. 2.4, we observe that

55 $$\begin{aligned} \langle I_{j} S_{k} \rangle \ge \langle S_{{k}} \rangle - \langle S_{{j}} \rangle - {\textbf{e}}\cdot \langle {\textbf{E}}_{j} \rangle - \langle R_{j} S_{k} \rangle , \end{aligned}$$

that

56 $$\begin{aligned} \frac{\textrm{d} {\langle R_{j} S_{k} \rangle }}{\textrm{d} {t}} \le \varvec{\nu }_{j} \cdot \langle {\textbf{E}}_{j} \rangle + \gamma _{j} \langle I_{j} S_{k} \rangle , \end{aligned}$$

and that

57 $$\begin{aligned} -\frac{\textrm{d} {\langle S_{{k}} \rangle }}{\textrm{d} {t}} \ge \lambda _{k \leftarrow j} \langle I_{j} S_{k} \rangle \end{aligned}$$

for any $$j\in {\mathcal {N}}(k)$$.

We also replicate some of the analysis from Sect. 3.2. We define ${\textbf{M}}_{k}$ as in (41) and we observe that equations (33a) and (33b) can be rearranged to give

58 $$\begin{aligned} \begin{bmatrix} \frac{\textrm{d} {\langle S_{{k}} \rangle }}{\textrm{d} {t}} \\ \frac{\textrm{d} {\langle {\textbf{E}}_{k} \rangle }}{\textrm{d} {t}} \end{bmatrix} = - {\textbf{M}}_{k} \begin{bmatrix} \displaystyle \sum _{j \in {\mathcal {N}}(k)} \lambda _{k \leftarrow j} \langle I_{j} S_{k} \rangle \\ \langle {\textbf{E}}_{k} \rangle \end{bmatrix}. \end{aligned}$$

Note that (58) is only valid because $\varvec{\varphi }_{k}$ depends only on $k$ not on the possible sources of infection. If this were not the case, then it would not be possible to collect the summation terms in the vector on the right hand side of (58).

Repeating the manipulations from Sect. 3.2, we find that

59 $$\begin{aligned} \varvec{\nu }_{j} \cdot \langle {\textbf{E}}_{j} \rangle = -\varvec{\nu }_{j}^T {\textbf{B}}_{j}^{-1} \varvec{\varphi }_{j} \frac{\textrm{d} {\langle S_{{j}} \rangle }}{\textrm{d} {t}} -\varvec{\nu }_{j}^T {\textbf{B}}_{j}^{-1} \frac{\textrm{d} {\langle {\textbf{E}}_{j} \rangle }}{\textrm{d} {t}}. \end{aligned}$$

Combining (56), (57), and (59), we find that

60 $$\begin{aligned} \frac{\textrm{d} {\langle R_{j} S_{k} \rangle }}{\textrm{d} {t}} \le -\varvec{\nu }_{j}^T {\textbf{B}}_{j}^{-1} \varvec{\varphi }_{j} \frac{\textrm{d} {\langle S_{{j}} \rangle }}{\textrm{d} {t}} - \varvec{\nu }_{j}^T {\textbf{B}}_{j}^{-1} \frac{\textrm{d} {\langle {\textbf{E}}_{j} \rangle }}{\textrm{d} {t}} - \frac{\gamma _{j}}{\lambda _{k \leftarrow j}} \frac{\textrm{d} {\langle S_{{k}} \rangle }}{\textrm{d} {t}}. \end{aligned}$$

Integrating from $t = 0$ and using the fact that $\langle R_{j} S_{k} \rangle (0) = 0$, we obtain an upper bound on $\langle R_{j} S_{k} \rangle$ that can be substituted into (55) and rearranged to obtain

61 $$\begin{aligned} \langle I_{j} S_{k} \rangle&\ge \frac{\lambda _{k \leftarrow j} +\gamma _{j}}{\lambda _{k \leftarrow j}} \langle S_{{k}} \rangle - (1-{\textbf{e}}\cdot \varvec{\varphi }_{j})\langle S_{{j}} \rangle (t) - \varvec{\mu }_{j}^T {\textbf{B}}_{j}^{-1} \left[ \varvec{\varphi }_{j} \langle S_{{j}} \rangle + \langle {\textbf{E}}_{j} \rangle \right] \nonumber \\&\quad -\varvec{\nu }_{j}^T {\textbf{B}}_{j}^{-1} \left[ \varvec{\varphi }_{j} \langle S_{{j}} \rangle (0) + \langle {\textbf{E}}_{j} \rangle (0) \right] - \frac{\gamma _{j}}{\lambda _{k \leftarrow j}}\langle S_{{k}} \rangle (0). \end{aligned}$$

Since it is also true that $\langle I_{j} S_{k} \rangle \ge 0$, we can use $[x]^+$ as defined in (23) to obtain a bound on $\langle I_{j} S_{k} \rangle$ analogous to (22). Substituting into (33a) then yields

62 $$\begin{aligned} \frac{\textrm{d} {\langle S_{{k}} \rangle }}{\textrm{d} {t}}&\le - \sum _{j \in {\mathcal {N}}(k)} \Big [(- \lambda _{k \leftarrow j} \varvec{\nu }_{j}^T {\textbf{B}}_{j}^{-1} \left[ \varvec{\varphi }_{j} \langle S_{{j}} \rangle (0) + \langle {\textbf{E}}_{j} \rangle (0) \right] - \gamma _{j}\langle S_{{k}} \rangle (0) \nonumber \\&\quad + (\lambda _{k \leftarrow j} + \gamma _{j}) \langle S_{{k}} \rangle - \lambda _{k \leftarrow j} (1-{\textbf{e}}\cdot \varvec{\varphi }_{j}) \langle S_{{j}} \rangle (t)\nonumber \\&\quad - \lambda _{k \leftarrow j} \varvec{\mu }_{j}^T {\textbf{B}}_{j}^{-1} \left[ \varvec{\varphi }_{j} \langle S_{{j}} \rangle + \langle {\textbf{E}}_{j} \rangle \right] \Big ]^{+}. \end{aligned}$$

We note that (62) depends only on the probabilities of nodes being susceptible or exposed. Hence, (62) can be coupled with (34b) to obtain a closed system. As in Sect. 2.4, we will use this closed system to show that In this case, however, we need to rearrange the system before we can apply the Kamke–Müller conditions.

Based on the forms of (62) and (34b), we define $\langle {\textbf{Q}}_{k} \rangle (t) = {\textbf{B}}_{k}^{-1} \big [ \varvec{\varphi }_{k}\langle S_{{k}} \rangle (t) + \langle {\textbf{E}}_{k} \rangle (t)\big ]$. We note that all entries of ${\textbf{B}}_{k}^{-1}$ are nonnegative and so $\langle {\textbf{Q}}_{k} \rangle$ is nonnegative. Rearranging to obtain $\langle {\textbf{E}}_{k} \rangle = {\textbf{B}}_{k} \langle {\textbf{Q}}_{k} \rangle - \varvec{\varphi }_{k} \langle S_{{k}} \rangle$ and substituting into (62) and (34b) then yields

63a $$\begin{aligned} \frac{\textrm{d} {\langle S_{{k}} \rangle }}{\textrm{d} {t}}&\le - \sum _{j \in {\mathcal {N}}(k)} \Big [(- \lambda _{k \leftarrow j} \varvec{\nu }_{j} \cdot \langle {\textbf{Q}}_{j} \rangle (0) - \gamma _{j}\langle S_{{k}} \rangle (0) + (\lambda _{k \leftarrow j} + \gamma _{j}) \langle S_{{k}} \rangle \nonumber \\&\qquad - \lambda _{k \leftarrow j} (1-{\textbf{e}}\cdot \varvec{\varphi }_{j}) \langle S_{{j}} \rangle (t) - \lambda _{k \leftarrow j} \varvec{\mu }_{j} \cdot \langle {\textbf{Q}}_{j} \rangle \Big ]^{+}, \end{aligned}$$

63b $$\begin{aligned} \frac{\textrm{d} {\langle {\textbf{Q}}_{k} \rangle }}{\textrm{d} {t}}&= -{\textbf{B}}_{k} \langle {\textbf{Q}}_{k} \rangle +\varvec{\varphi }_{k} \langle S_{{k}} \rangle . \end{aligned}$$

System (63) is a system of differential inequalities and equations; as in Sect. 2.4, we now consider the relationship between the true solutions $\langle S_{{k}} \rangle$ and $\langle {\textbf{Q}}_{k} \rangle$ and the approximate solutions $\langle S^{*}_{{k}} \rangle$ and $\langle {\textbf{Q}}^{*}_{k} \rangle$ that satisfy the equivalent of (63) where the inequality in (63a) is replaced with an equation. Since the off-diagonal elements of ${\textbf{B}}_{k}$ are all nonpositive, since $1-{\textbf{e}}\cdot \varvec{\varphi }_{k} \ge 0$, and since the elements of $\varvec{\varphi }_{k}$ and $\varvec{\mu }_{k}$ are all nonnegative, this system will satisfy the Kamke–Müller conditions and be cooperative. Hence, we can again apply Lemma 1 from Simon and Kiss (2018) to conclude that $\langle S^{*}_{{k}} \rangle (t) \ge \langle S_{{k}} \rangle (t)$ and that $\langle {\textbf{Q}}^{*}_{k} \rangle (t) \ge \langle {\textbf{Q}}^{*}_{k} \rangle (t)$ for all $k$ and for all *t*.

While $\langle {\textbf{Q}}_{k} \rangle$ is a useful theoretical construct, we will generally formulate and solve the SEIR rooted-tree approximation using $\langle {\textbf{E}}_{k} \rangle$ rather than $\langle {\textbf{Q}}_{k} \rangle$. Using stars to indicate approximate solutions as previously, we use (62) and (34) to obtain the following system as the SEIR rooted-tree approximation:

64a $$\begin{aligned} \frac{\textrm{d} {\langle S^{*}_{{k}} \rangle }}{\textrm{d} {t}}&=- \sum _{j \in {\mathcal {N}}(k)} \Big [(- \lambda _{k \leftarrow j} \varvec{\nu }_{j}^T {\textbf{B}}_{j}^{-1} \left[ \varvec{\varphi }_{j} \langle S^{*}_{{j}} \rangle (0) +\langle {\textbf{E}}^{*}_{j} \rangle (0) \right] - \gamma _{j}\langle S^{*}_{{k}} \rangle (0) \nonumber \\&\quad + (\lambda _{k \leftarrow j} + \gamma _{j}) \langle S^{*}_{{k}} \rangle - \lambda _{k \leftarrow j} (1-{\textbf{e}}\cdot \varvec{\varphi }_{j}) \langle S^{*}_{{j}} \rangle (t)\nonumber \\&\quad - \lambda _{k \leftarrow j} \varvec{\mu }_{j}^T {\textbf{B}}_{j}^{-1} \left[ \varvec{\varphi }_{j} \langle S^{*}_{{j}} \rangle +\langle {\textbf{E}}^{*}_{j} \rangle \right] \Big ]^{+}, \end{aligned}$$

64b $$\begin{aligned} \frac{\textrm{d} {\langle {\textbf{E}}^{*}_{k} \rangle }}{\textrm{d} {t}}&= -\varvec{\varphi }_{k} \frac{\textrm{d} {\langle S^{*}_{{k}} \rangle }}{\textrm{d} {t}} - {\textbf{B}}_{k} \langle {\textbf{E}}^{*}_{k} \rangle , \end{aligned}$$

64c $$\begin{aligned} \frac{\textrm{d} {\langle I^{*}_{k} \rangle }}{\textrm{d} {t}}&=-(1 - {\textbf{e}}\cdot \varvec{\varphi }_{k}) \frac{\textrm{d} {\langle S^{*}_{{k}} \rangle }}{\textrm{d} {t}} - \gamma _{k} \langle I^{*}_{k} \rangle + \varvec{\mu }_{k} \cdot \langle {\textbf{E}}^{*}_{k} \rangle . \end{aligned}$$

Just as (28) is equivalent to (9) for a rooted tree, we can show that (64) is equivalent to (53) for a rooted tree. To see this, we again use the fact that $\langle S^{*}_{{j}} \rangle (t) \ge \langle S^{*}_{{k}} \rangle (t)$ for any $j\in {\mathcal {N}}(k)$ other than $j= p(k)$, and we also use the fact that ${\textbf{B}}_{k}^{-T} \varvec{\mu }_{k} + {\textbf{B}}_{k}^{-T} \varvec{\nu }_{k} = {\textbf{e}}$. Given that $\langle S^{*}_{{k}} \rangle (t)$ is a decreasing function of *t*, it follows from these observations that

65 $$\begin{aligned} \varvec{\nu }_{j}^T {\textbf{B}}_{j}^{-1} \varvec{\varphi }_{j} \langle S^{*}_{{j}} \rangle (0) + (1-{\textbf{e}}\cdot \varvec{\varphi }_{j}) \langle S^{*}_{{j}} \rangle (t) + \varvec{\mu }_{j}^T {\textbf{B}}_{j}^{-1} \varvec{\varphi }_{j} \langle S^{*}_{{j}} \rangle (t) \ge \langle S^{*}_{{j}} \rangle (t) \end{aligned}$$

and hence the term inside the square brackets in (64a) will be nonpositive whenever $j \ne p(k)$. As a result, (64) will yield exact solutions for rooted trees without it being necessary to compute the parent of each node.

**Fig. 4:**
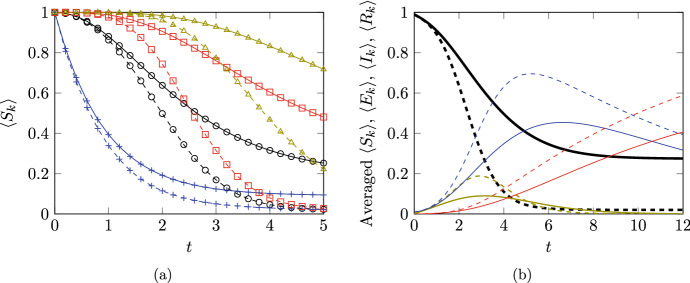
Comparisions of the rooted-tree approximation in (64) with simulation results from the average of $10^5$ Gillespie algorithm simulations of the full stochastic SEIR model for an Erdős–Rényi random graph with 100 nodes and probability of connection 0.05. Subfigure (**a**) shows $\langle S_{{k}} \rangle$ for four different nodes: results from the rooted-tree approximation are shown as continuous lines and results from Gillespie simulations are shown as dashed lines; different nodes are distinguished using different colours and marker styles. Subfigure (**b**) show $\langle S_{{k}} \rangle$ (very thick black lines), $\langle E_{k} \rangle$ (thick olive lines), $\langle I_{k} \rangle$ (medium thickness blue lines) and $\langle R_{k} \rangle$ (thin red lines) averaged over all nodes in the network: results from the rooted-tree approximation are shown as continuous lines and results from Gillespie simulations are shown as dashed lines. Parameters used are $\lambda _{} = 1$, ${\varphi }_{} = 0.8$, ${\mu }_{} = 1.2$, ${\nu }_{} = 0.05$, and $\gamma _{} = 0.1$. There is a single node that is infectious at $t=0$ and all other nodes are susceptible

Figure 4 is analogous to Fig. 2 and it enables equivalent conclusions to be drawn. Figure 4 shows comparisons of the rooted-tree approximation (64) with estimates of the true solution obtained from averaging $10^5$ simulations using the Gillespie algorithm for an ER random graph. Code is available at https://github.com/cameronlhall/rootedtreeapprox and the ER graph used to generate Fig. 4 is different from the ER graph used in Fig. 2.

From Fig. 4a we verify that the rooted-tree approximation gives an upper bound on $\langle S_{{k}} \rangle$ for the nodes $k$ illustrated. From Fig. 4b, we see that there is a reasonably large difference between the true solution (dashed lines) and the rooted-tree approximation (continuous lines) and so once again the bounds provided by (64) are not generally very tight.

## Discussion and conclusions

In this paper, we have developed and analysed a new approximation method, the rooted-tree approximation, that can be applied to SIR and generalised SEIR models on networks. In the case of a tree with a unique initially-infected node, our approximation is exact and leads to a partially-decoupled system of linear differential equations for the node-state probabilities. As demonstrated in Sect. 2.3, we can obtain explicit closed-form solutions for the node state probabilities for SIR models and, in theory, equivalent results can also be obtained for SEIR models.

Since the pair-based and message-passing approximations are both exact on *all* trees (not just rooted trees) but closed-form solutions for these are not well known, it is instructive to compare our system (9) with equivalent rooted tree simplifications of the pair-based SIR approximation in Sharkey et al. (2015) and the message-passing SIR approximation in Karrer and Newman (2010). For the pair-based approximation (*e.g.*, system (3) in Sharkey et al. (2015)), we find that we can use proof by induction from the leaves to the root to show that $\langle I_{k} S_{p(k)} \rangle = 0$ on a rooted tree. Subsequently, we can use the fact that $\langle S_{p(k)} S_{k} \rangle = \langle S_{{k}} \rangle$ to convert the remaining equations of the pair-based approximation into a linear system equivalent to (9).

For the message-passing model in Karrer and Newman (2010) applied to a rooted tree, we can work from the leaves to the root to show that $H^{p(k)\leftarrow k} = 0$ and then work back out from the root to the leaves to obtain expressions for $H^{k\leftarrow p(k)}$ that are analogous to an integrated form of our system (9). As a result, we find that the explicit solutions in (13) could have been obtained from the pair-based or message-passing approximations; while we believe that this is the first time that these explicit solutions have been reported, they are consistent with—and theoretically obtainable from—established results in the existing literature.

One important feature of our rooted-tree approximation is that it provides upper bounds on $\langle S_{{k}} \rangle$ at every node. This is an important strength of our method since it provides a contrast from other methods that yield lower bounds on $\langle S_{{k}} \rangle$. A promising avenue for further research would be to combine the rooted-tree approximation with other approximations in order to obtain better estimates of node-state probabilities. Such hybrid approximations are likely to be more practical than the rooted-tree approximation because the bounds on $\langle S_{{k}} \rangle$ are rarely very tight. As we see from Figs. 2 and 4, there are often large differences between the node-state probabilities obtained from the rooted-tree approximation and estimates of the true node-state probabilities based on Gillespie algorithm simulations.

A limitation of our rooted-tree methodology is that it relies on two key assumptions: firstly, that there can be no return to a susceptible state following infection, and secondly that there can only be one class of infectious state. Both of these assumptions are necessary in order to express $\langle I_{p(k)} S_{k} \rangle$, and hence the rate of infection, in terms of a linear combination of the node-state probabilities and $\langle R_{p(k)} S_{k} \rangle$ for rooted trees. One direction for further research would be to explore whether the rooted-tree approximation can be extended to SIRS and SEIRS models or SIR models with multiple infectious states. Perhaps this would involve developing new approximations that are not exact on rooted trees but would still provide a consistent upper bound on $\langle S_{{k}} \rangle$, analogous to the $W(x,y) = \min (x,y)$ approximation for SIS models introduced in Simon and Kiss (2018).

Overall, the rooted-tree approximation presented in this paper is a new way of analysing SIR and SEIR dynamics on networks that has advantages and disadvantages over existing methods. The principal strengths of the rooted-tree approximation are that it is simple (leading to a cooperative, piecewise-linear system of equations for node-state probabilities), that it yields exact closed-form solutions in certain situations, and that it yields upper bounds on $\langle S_{{k}} \rangle$ in contrast with the lower bounds provided by other approximations. The principal weakness of the rooted-tree approximation is that the bounds on $\langle S_{{k}} \rangle$ are not very tight unless the underlying network is very close to being a tree with a single initially-infected node. Despite this limitation, the simplicity of the rooted-tree approximation means that it has the potential to be a useful tool in developing new computational methods for analysing contagion dynamics on networks.

## References

[CR1] Bame N, Bowong S, Mbang J, Sallet G, Tewa J-J (2008). Global stability analysis for SEIS models with $$n$$ latent classes. Math Biosci Eng.

[CR2] Berman A, Plemmons RJ (1994) Nonnegative Matrices in the Mathematical Sciences. Society for Industrial and Applied Mathematics. 10.1137/1.9781611971262

[CR3] Biggs NL (2002). Discrete mathematics.

[CR4] Brauer F, Castillo-Chavez C, Feng Z (2019). Mathematical models in epidemiology.

[CR5] Cator E, Donnelly P, Mieghem PV (2018) Reply to ‘Comment on ‘Nodal infection in Markovian susceptible-infected-susceptible and susceptible-infected-removed epidemics on networks are non-negatively correlated”. Physical Review E 98. 10.1103/physreve.98.026302 10.1103/PhysRevE.98.026302 30253615

[CR6] Cator E, Mieghem PV (2012) Second-order mean-field susceptible-infected-susceptible epidemic threshold. Physical Review E 85. 10.1103/physreve.85.056111 10.1103/PhysRevE.85.056111 23004825

[CR7] Cator E, Mieghem PV (2014). Nodal infection in Markovian susceptible-infected-susceptible and susceptible-infected-removed epidemics on networks are non-negatively correlated. Phys Rev E.

[CR8] Cunniffe NJ, Stutt ROJH, van den Bosch F, Gilligan CA (2012). Time-dependent infectivity and flexible latent and infectious periods in compartmental models of plant disease. Phytopathology.

[CR9] Danon L, Ford AP, House T, Jewell CP, Keeling MJ, Roberts GO, Ross JV, Vernon MC (2011). Networks and the epidemiology of infectious disease. Interdiscip Perspect Infect Dis.

[CR10] Diekmann O, Heesterbeek JAP, Roberts MG (2010). The construction of next-generation matrices for compartmental epidemic models. J R Soc Interface.

[CR11] Donnelly P (1993). The correlation structure of epidemic models. Math Biosci.

[CR12] Guo H, Li MY, Shuai Z (2012). Global dynamics of a general class of multistage models for infectious diseases. SIAM J Appl Math.

[CR13] Kang H, Sun M, Yu Y, Fu X, Bao B (2020). Spreading dynamics of an SEIR model with delay on scale-free networks. IEEE Trans Netw Sci Eng.

[CR14] Karrer B, Newman MEJ (2010) Message passing approach for general epidemic models. Physical Review E 82. 10.1103/physreve.82.016101 10.1103/PhysRevE.82.016101 20866683

[CR15] Kiss IZ, Miller JC, Simon PL (2017) Mathematics of Epidemics on Networks, Springer-Verlag GmbH, https://www.ebook.de/de/product/33441347/istvan_z_kiss_joel_c_miller_peter_l_simon_mathematics_of_epidemics_on_networks.html

[CR16] Liu Q, Li T, Sun M (2017). The analysis of an SEIR rumor propagation model on heterogeneous network. Phys A.

[CR17] Miller JC, Kiss IZ (2014). Epidemic spread in networks: existing methods and current challenges. Math Model Natural Phenomena.

[CR18] Newman M (2018) Networks, Oxford University Press, 2nd ed., https://www.ebook.de/de/product/32966014/mark_newman_networks.html

[CR19] Pastor-Satorras R, Castellano C, Mieghem PV, Vespignani A (2015). Epidemic processes in complex networks. Rev Mod Phys.

[CR20] Petersen KB, Pedersen MS (2012) The matrix cookbook, http://www2.compute.dtu.dk/pubdb/pubs/3274-full.html. Version 20121115

[CR21] Sharkey KJ, Kiss IZ, Wilkinson RR, Simon PL (2015). Exact equations for SIR epidemics on tree graphs. Bull Math Biol.

[CR22] Sharkey KJ, Wilkinson RR (2015). Complete hierarchies of SIR models on arbitrary networks with exact and approximate moment closure. Math Biosci.

[CR23] Simon PL, Kiss IZ (2018). On bounding exact models of epidemic spread on networks. Dis Continuous Dynam Syst- B.

[CR24] Van Mieghem P, Omic J, Kooij R (2009). Virus spread in networks. IEEE/ACM Trans Netw.

[CR25] Wilkinson RR, Sharkey KJ (2014) Message passing and moment closure for susceptible-infected-recovered epidemics on finite networks. Physical Review E 89. 10.1103/physreve.89.022808 10.1103/PhysRevE.89.022808 25353535

